# The nonmedical use of prescription ADHD medications: results from a national Internet panel

**DOI:** 10.1186/1747-597X-2-32

**Published:** 2007-10-31

**Authors:** Scott P Novak, Larry A Kroutil, Rick L Williams, David L Van Brunt

**Affiliations:** 1 RTI International, 3040 Cornwallis Road, Research Triangle Park, NC 27709, USA; 2 Eli Lilly and Company, Lilly Research Laboratories, Indianapolis, IN 46285, USA

## Abstract

**Background:**

Emerging evidence suggests that nonmedical use (NMU) of prescription attention deficit/hyperactivity disorder (ADHD) medications is rising, but many previous investigations have used clinical or regionally based samples or limited their investigations to stimulants rather than to medications specifically used to treat ADHD. Using an Internet-based epidemiological survey, this paper advances understanding of the prevalence and correlates of NMU of medications used to treat ADHD, sources of diverted medications, motivations for use, and consumption patterns.

**Methods:**

The study used a self-administered Internet survey of civilian, noninstitutionalized adults (N = 4,297) aged 18 to 49 in the United States. National-level estimates were created using propensity scoring methods and weighting procedures using data from three nationally representative probability surveys: a random-digit dialed telephone survey, the current U.S. Census, and the National Survey on Drug Use and Health (NSDUH).

**Results:**

Past-year prevalence of NMU of ADHD medications was approximately 2%, with 4.3% reported among those aged 18 to 25 and 1.3% among those aged 26 to 49. Most respondents reporting NMU used on multiple occasions. Receipt of medications for ADHD was a significant correlate of past-year NMU, though most nonmedical users never had a prescription. Among persons who had never been prescribed medication to treat ADHD, friends or family members were the most common source. Productivity was the most frequently endorsed reason for NMU. Alcohol was the substance most commonly used in combination with ADHD drugs.

**Conclusion:**

Because most prescription ADHD medications currently are highly regulated, policy options for supply-side reduction of nonmedical use may include identifying those medications with lower abuse liability for inclusion on insurance formularies. Patient and physician education programs also may be useful tools to heighten awareness of intentional and unintentional diversion of ADHD medications for nonmedical purposes.

## Background

Clinician awareness of the presence and burden of attention deficit/hyperactivity disorder (ADHD) has increased in the United States and internationally [1-4]. In parallel, prescribing of medication for ADHD has increased over the past several years, particularly for adults [5,6]. Thus, the number of youth and adult patients receiving pharmacologic ADHD treatment is growing, and treatment includes a diverse range of stimulant and nonstimulant medications. As the number of prescriptions increases, the availability of these medications for diversion to nonmedical use (NMU) also increases.

What is largely known about NMU of ADHD drugs is derived from data sources that may not focus specifically on ADHD medications or from samples with limited generalizability. Most of these investigations have grouped a variety of prescription stimulants (e.g., methamphetamine, ADHD medications, anorectic drugs) as a broad therapeutic class. A study that specifically identified the NMU of prescription ADHD stimulants was conducted by Kroutil and colleagues in 2006 using the 2002 National Survey on Drug Use and Health (NSDUH) [7]. Except for methamphetamine, however, the 2002 NSDUH asked about NMU of specific stimulants only for the lifetime period. Therefore, measurement of past-year NMU of ADHD stimulants in the study was limited to persons who had never used any other types of stimulant nonmedically. Based on these data, the past-year prevalence of NMU of only ADHD stimulants was higher among young adults aged 18 to 25 (1.3%) than among persons aged 12 to 17 (0.9%) or persons aged 26 or older (0.1%). The demographic correlates also differed between stimulants in general and ADHD stimulants. This suggests that information about NMU of prescription stimulants may not be applicable to NMU of ADHD-specific medications. However, limiting measurement of past-year NMU of ADHD drugs to persons who exclusively misused these medications likely underestimated the true population prevalence, as it would have excluded persons who were nonmedical users (NMUs) of both ADHD stimulants and other stimulants in the past year. Further, NSDUH captures information on only a small subset of stimulant medications used to treat ADHD and excludes the newer classes of nonstimulant ADHD medications. Even for ADHD medications that are considered to have lower abuse potential, information about their use outside of medical supervision or in doses exceeding a treatment regimen would constitute problematic use due to potential side effects or drug interactions.

Other studies have observed higher rates of stimulant misuse among those aged 18 to 25, college students, and polydrug users [8-11]. Past studies examining polydrug use typically define use of multiple drugs based on a summary of the number of different substances used at any time in the past year. One notable exception that examined the co-occurrence of use of multiple substances was a study conducted by McCabe and colleagues [11]. However, the sample was limited to college students at a single university, and only the co-occurrence between alcohol and prescription medications, including stimulants, was investigated. More specific information is needed about consumption patterns using a more diverse sample to examine whether users are combining multiple drugs within a single drug-using episode, and if so, which drugs are used together with ADHD medications. This is significant given the potential adverse health risks associated with particular drug combinations involving ADHD medications [12].

Compared to studies examining the prevalence and correlates of nonmedical ADHD drug use, fewer studies have specifically examined dimensions of access and motivation for NMU of ADHD medications. McCabe et al. [13] reported that in a sample of middle and high school students in the Midwest, over 23% of those with a prescription for an ADHD medication were approached to sell, trade, or give away their medications. This finding is confirmed in the 2005 NSDUH, which found that most persons who used prescription stimulants (excluding methamphetamine) nonmedically in the past year received them from friends or relatives for free [14]. Some studies have examined associations between NMU and psychological factors, including ADHD status [15,16] to identify possible motivations for use. For example, a clinical case-control study by Wilens and colleagues [17] based on ADHD diagnosis (N = 186) found that 36% of the sample reported use for self-medication, 25% used ADHD medications to get high, and 39% had unknown motivation. A community-based study at a single university found that nearly 25% of those with ADHD reported use of their medications for recreational purposes [18]. Yet, much of the literature points to NMU of prescription stimulants for performance enhancement. Teter et al. [19], in a study at a large, midwestern university, found that prescription stimulants were used primarily for performance enhancement, although use for feelings of euphoria (e.g., getting high) also was noted. These studies, while primarily limited to regional populations, suggest that peers are a common source of diverted medications and that performance enhancement or self-medication are important motivations for NMU.

Some of these investigations used large, epidemiological surveillance systems (e.g., NSDUH) that offer considerable breadth but may lack sufficient depth for examining, in detail, more focused topics such as NMU of ADHD medications. In contrast, a small but insightful number of investigations focusing on patterns of diversion and motivation for NMU of ADHD medications have relied on small, regionally based or clinical samples [20,21]. The costs and resources needed to recruit a sample large enough to support reliable estimates of NMU of ADHD medications could be an important barrier to designing new national-level and international-level studies with specific foci on ADHD medication NMU and diversion.

Internet surveys have emerged as an alternative data collection method when in-person or telephone surveys could be costly or time-consuming. Berrens et al. [22] list several features of Internet surveys that make this design well-suited for a study of diverted ADHD medication use, including (1) low marginal costs because no interviewer labor is involved in screening or interviewing, (2) the ability to use visual cues (e.g., pictures of medications) via the Web to aid respondent recall, (3) rapid assembly of samples, and (4) rapid data collection from respondents with less prevalent characteristics of interest [7,23]. Concerns that have been cited about some Internet surveys focus on the use of nonprobability samples [24]. However, these concerns may be partly offset by an increased level of access to the Internet by a broader range of socioeconomic and demographic groups, particularly younger adults [25,26], a subpopulation with higher rates of NMU of prescription drugs. Internet recruitment can use a probability sample to select respondents who then complete the interview via a Web-based data collection system. However, Internet market research panels have emerged as an avenue for recruitment because a sample may be drawn from a preexisting opt-in panel of possible respondents that have agreed to complete regular online surveys. In this regard, "opt-in panel" refers to a group of respondents that have agreed to participate in multiple surveys, rather than a single group of participants in a longitudinal study. Two techniques that also have been used to increase the confidence in findings from this approach include targeted recruitment based on known demographic characteristics of panel participants and use of propensity weighting methods to approximate the results that would have been obtained from a probability-based survey [27]. Yet, Internet panel data should be carefully evaluated for bias, and regarded as an initial step in producing estimates that shed preliminary insight into the nature and scope of an emergent issue, or one about which little is known, such as prescription drug abuse.

In light of these issues, this diversion study reports findings using an existing opt-in Internet sample recruited from the entire United States for the following purposes: (1) provide estimates of NMU of ADHD medications by identifying specific medications used in the treatment of ADHD rather than using a general question about "prescription stimulants" commonly used, (2) identify the subpopulations (e.g., those with ADHD) at greatest risk for NMU, (3) identify important sources of diverted medications, (4) examine motivations for NMU, and (5) estimate the patterns of alcohol and other drug use in combination with ADHD medications.

## Methods

### Study sample and procedures

Participants were drawn from Harris Interactive's Harris Poll Online (HPOL) panel, which consists of several million members internationally who consented to be contacted for public opinion surveys administered through the Internet. Members are added to the database after they have completed a two-stage registration process: first by signing on to the HPOL Web site and then by responding to a confirmation e-mail message providing a confidential password. Data are collected through an encrypted, password-protected Web portal. Eligible panelists were noninstitutionalized civilian adults aged 18 to 49 living in the United States (50 states and the District of Columbia). A minimum age of 18 was chosen because the time and costs of obtaining active parental consent for minors likely would have offset the benefits of online data collection. The maximum age was set at 49 to increase the efficiency of the sampling design, taking into account the low prevalence of NMU of prescription drugs among persons aged 50 or older [23] and the number of respondents who would be needed to generalize to this age group. Moreover, the NSDUH, which was used as a benchmark to post-stratify the data, collapses ages 50 to 65 into a single category rather than reporting age-specific data for this range. The sample size was determined from prevalence rates of NMU of ADHD stimulants estimated from Kroutil et al. [7]. These estimates provided sample sizes for three targets based on two broad age groupings (18 to 25 and 26 to 49), gender, and NMU of ADHD stimulants (lifetime and past 3 years). These procedures favored the representation of whites, males, and young adults to ensure adequate numbers of nonmedical users (NMUs) in the final sample.

The survey was conducted during August 2005. Study enrollment and consent involved a two-part process. First, e-mail invitations describing the study were sent to panel members who initially met eligibility criteria according to data in the Harris Interactive (HI) database on panel members' age group and country of residence. These criteria were based on the NSDUH, which collects information from residents of households, noninstitutional group quarters (e.g., shelters, rooming houses, dormitories), and civilians living on military bases. Persons excluded from the survey include homeless persons who do not use shelters, active-duty military personnel, and residents of institutional group quarters, such as prisons and long-term hospitals. Respondents completed a short screener that verified these characteristics. The screener also asked about respondents' military status, living situation, and substance use history, including NMU of ADHD medications; target groups were filled according to screening responses. For consistency with the NSDUH, active-duty military personnel and residents of institutional group quarters (e.g., long-term care facilities) were excluded from the study. Eligible respondents (i.e., those whose target groups had not been filled) were routed to a more detailed consent statement for participation in the full survey. Respondents who otherwise would have been eligible but whose target groups had already been filled did not complete the full survey. The survey took an average of 20 minutes to complete if respondents reported NMU.

A total of 295,414 e-mail invitations were sent out, and 11,200 responses were received, regardless of outcome, or about 3.8% of the invitations. Of the 11,200 HPOL members who responded, 4,541 met the eligibility criteria and were needed for the study (i.e., their target groups had not yet been filled). Among this latter group, 4,297 (94.6%) completed the survey, and 244 (5.4%) declined to participate or did not complete the survey by the close of data collection.

### Weighting procedures

We employed a two-part methodology to adjust for Internet responses relative to the U.S. population of adults aged 18 to 49. First, propensity scoring methods were used to weight the data to approximate results for a probability-based telephone survey. Second, the data were further weighted to match the U.S. target population distribution by general demographic characteristics and to match the distribution of past-month cigarette use and past-month binge alcohol use estimated from 2003 NSDUH, which were the most current publicly available data during the data processing phase of this study, in 2005 [28]. These procedures are discussed in further detail below.

The first step in the weighting procedure involved propensity scoring to reduce the bias attributable to collecting data from only HPOL members. The propensity score was included to adjust for self-selection into the online population and the panel, as well as for survey nonresponse that may not be explained by demographic differences. HI periodically conducts parallel telephone and Web surveys to measure attitudinal and behavioral variables that predict one's propensity to be online; the telephone survey is based on random-digit dialing (RDD) probability sampling. Using the combined telephone and Web surveys, HI created a propensity score model [27] for online survey response. Weights were created so that the weighted distribution of the propensity score for Web respondents was matched to the distribution for the RDD telephone respondents. This approach caused the weighted distribution of the attitudinal and behavioral variables of Web respondents to be aligned with those of the RDD telephone respondents. In short, the propensity score adjustment may make the Internet survey estimates more comparable to those obtained from an RDD telephone survey. It is similar to adjustments made in clinical and observational studies to ensure that groups are balanced on potentially confounding characteristics. In this case, the effect of confounding by selection into the Internet panel is adjusted using information from an RDD telephone survey. The weighting targets for the telephone survey were derived from the Current Population Survey conducted annually by the U.S. Census.

The second step was that the propensity score-adjusted weights that RTI obtained from HI underwent further calibration using the generalized exponential model [29] using data from the 2003 NSDUH; the 2003 NSDUH had a weighted household screening response rate of 90.7% and a weighted interview response rate of 77.4% [30]. We used past-month cigarette use and past-month binge alcohol use from NSDUH as a benchmark in creating final weights to make appropriate inferences to the noninstitutionalized U.S. adult population aged 18 to 49. Both of these variables are likely related to a respondent's probability of substance use and a respondent's likelihood of being in a Web panel [31]. Age, gender, and highest education also were included in the weight adjustment to bring the weighted distribution of the Internet respondents in line with the U.S. target population. This strategy further aligned the Internet sample with the large, nationally representative NSDUH on factors related to NMU of prescription medications. Although a detailed accounting of the propensity model is beyond the scope of the current manuscript, this additional supplemental information is freely available by contacting the primary author.

We also evaluated bias by comparing estimates in our survey with those for analogous variables in the 2005 NSDUH. Correspondence between the 2005 Diversion II estimates and the 2005 NSDUH suggests that the propensity model developed using 2003 NSDUH data, a follow-up telephone survey, and the U.S. census successfully reduced bias in the estimates, thus providing a large degree of confidence in the resulting estimates.

### Sample characteristics and correspondence with the 2005 NSDUH

Table 1 provides a demographic breakdown of the study sample, including unweighted and weighted percentages. Because our procedures targeted specified numbers of NMUs, young adults and whites were overrepresented in the survey; however, the final analysis weights of young adults and whites reflect their representation in the U.S. population aged 18 to 49.

**Table 1:** Sample characteristics of 2005 ADHD diversion study

	**Sample *n***	**Sample Percent**	**Weighted Percent**^a^	**SE**
**Age**				
18–25	3,307	76.96	24.19	1.10
26–49	990	23.04	75.81	1.10
**Sex**				
Male	1,857	43.22	49.46	1.95
Female	2,440	56.78	50.54	1.95
**Race/ethnicity**				
White, not Hispanic	3,294	76.66	65.59	1.95
Black, not Hispanic	278	6.47	12.28	1.23
Other, not Hispanic	354	8.24	6.75	1.04
Hispanic	371	8.63	15.37	1.69
**Education**				
Some high school	399	9.29	16.55	2.24
High school graduate	952	22.15	31.06	1.70
Some college	1,966	45.75	26.86	1.43
College graduate	980	22.81	25.53	1.47
**Student status (ages 18–25)**				
Student	1,646	49.77	39.50	1.58
Nonstudent	1,661	50.23	60.50	1.58
**Insurance**				
Private/public	3,351	79.26	75.71	2.01
None	877	20.74	24.29	2.01
**ADHD status**				
No lifetime diagnosis	4,069	94.69	96.85	0.40
Lifetime diagnosis, no medications in past year	134	3.12	1.67	0.28
Medications in past year^b^	90	2.09	1.41	0.28

ADHD = attention deficit hyperactivity disorder.

^a ^Weighted to the U.S. population of noninstitutionalized civilian adults aged 18–49.

^b ^Excludes four respondents who reported fabricating symptoms or obtaining medications from a doctor "who didn't ask too many questions" in the past year.

Table 2 compares estimates from the 2005 diversion study and the 2005 NSDUH, including significance tests and effect sizes. We present effect sizes using Cohen's d [32] because the combined samples of the NSDUH (N = 32,104) and diversion study (N = 4,297) had sufficiently high power to classify even the smallest of differences as statistically significant. Measures in Table 2 were not used in our weight calibrations. Therefore, they indicate how well our weighting procedures yielded estimates that are comparable to a large, national probability survey. Of the drug use items common to both studies, only the prevalence estimate for lifetime NMU of Cylert (pemoline) was significantly different, though the effect size suggests that this difference was relatively small.

**Table 2:** Selected lifetime nonmedical use of prescription and illicit drugs, ADHD diversion study, National Survey of Drug Use and Health, 2005

	**Lifetime Use**					
	
	**2005 Diversion**		**2005 NSDUH**			
			
	***n *= 4,297**		***n *= 32,104**			
	**%**^a^	**SE**	**%**^a^	**SE**	***P***^b^	**ES**^c^
**Illicit Drugs**						
Marijuana	57.54	1.87	53.66	0.54	0.05	0.078
Cocaine	23.47	2.01	20.23	0.37	0.11	0.081
Methamphetamine	8.63	1.50	5.83	0.21	0.06	0.119
Supraval^d^	0.11	0.05				
**Prescription stimulants**^e^						
Prescription diet pills^f^	3.95	0.90	3.46	0.15	0.59	0.027
Ritalin or methylphenidate	4.20	0.76	2.67	0.12	0.05	0.095
Cylert or pemoline	0.31	0.10	0.08	0.03	0.03	0.081
Dexedrine, Dextrostat, or Dexampex	1.67	0.65	0.75	0.06	0.16	0.107
Dextroamphetamine	0.67	0.26	0.20	0.03	0.08	0.105
**Prescription stimulants**^e^						
Preludin or phenmetrazine	0.49	0.25	0.16	0.03	0.20	0.083
Any Adderall	2.06	0.21	n/a			
Adderall	1.58	0.19	n/a			
Adderall XR	0.80	0.14	n/a			
Concerta	0.87	0.30	n/a			
Any ADHD stimulants^g^	7.01	0.87	n/a			
**ADHD Nonstimulants**						
Modafanil	0.42	0.24	n/a			
Strattera	0.53	0.25	n/a			
**Any ADHD medications**^h^	**7.07**	**0.87**	**n/a**			

ADHD = attention deficit hyperactivity disorder; n/a = Estimate not available from the 2005 NSDUH; NSDUH = National Survey of Drug Use and Health.

^a ^Weighted to the U.S. population of noninstitutionalized civilian adults aged 18 to 49.

^b ^*P *value derived from chi-square test in SUDAAN.

^c ^Effect size, determined by the difference in prevalence estimates between the 2005 Diversion Study and the 2005 NSDUH. In the social sciences, an effect size is considered to be "small" when it is about 0.10, "medium" when it is about 0.30, and "large" when it is about 0.50, based on Cohen's d (1988) [32].

^d ^Fictitious drug used to assess reporting bias.

^e ^Nonmedical use.

^f ^Examples given in both studies were amphetamines, Benzedrine, Biphetamine, Fastin, or phentermine. Because some of these medications may be used to treat ADHD (e.g., amphetamines), respondents in the 2005 Diversion Study who reported lifetime nonmedical use of "prescription diet pills" were subsequently asked separate questions about nonmedical use of (a) amphetamines; (b) Benzedrine; (c) Biphetamine; (d) Fastin, or phentermine; or (e)some other prescription diet pill.

^g ^ADHD stimulants referred to any of the following: amphetamines or dextroamphetamine (including any form of Adderall, Biphetamine, Dexedrine, Dextrostat, or Dexampex); any form of methylphenidate or dexmethylphenidate (including Ritalin, Concerta, Methylin, or Focalin); or Cylert or pemoline.

^h ^Nonmedical use of any ADHD stimulants or nonstimulants in the period of interest.

### Instrument and measures

Demographic and substance use items were based directly on public-domain NSDUH questions to establish comparability in the estimates between the two studies. NMU of prescription drugs was defined as use without a prescription or for the feeling or experience; examples of exact question wordings in the diversion study are shown in the appendix. Consistent with NSDUH, respondents were asked about the most recent NMU, including NMU in the 12-month period prior to taking the survey (i.e., "past year").

NSDUH asks about lifetime NMU of a subset of ADHD medications but does not ask specifically about most recent NMU of ADHD medications. Therefore, we adapted NSDUH questions to ask about the most recent NMU of a wider range of ADHD medications. Similar to NSDUH, pictures of prescription pills were used to facilitate recall. For comparability with NSDUH, we asked respondents about NMU of brand-name drugs followed by their generic equivalents, or about NMU of specific brand-name drugs only. In addition, we asked respondents about NMU of a fictitious drug (Supraval) to gauge the potential for indiscriminate reporting of NMU [33]. After completing the medication module, respondents were asked the number of days in the past year they used any ADHD medication.

NSDUH also does not capture information on ADHD symptomatology. Therefore, we created items to capture whether respondents reported ever receiving a diagnosis of ADHD from a medical professional *and *whether they ever received prescription medication for their condition. Among respondents who reported that they had received prescriptions for ADHD in the past year, we excluded four who reported that they obtained some medications by misrepresenting their symptoms or by going to doctors with lenient prescribing practices. We defined remaining respondents who received prescriptions for their ADHD in the past year as having "legitimate" prescriptions because they did not report NMU, or if they did, they did not obtain their medications fraudulently from physicians.

Using existing items from prior studies [28,34], respondents with past-year NMU of ADHD medications were asked to report ways in which they obtained these drugs in that period. In addition, respondents who reported past-year NMU were asked about drugs they used at the same time or within a couple of hours of their NMU of ADHD medications. Past-year NMUs also were asked the primary reason for misusing these ADHD medications [35] (see the appendix for question wording). Of note was that student status refers to current enrollment in high school, college, technical school, or advanced graduate studies.

### Analysis

Descriptive statistics include prevalence estimates (percentages), the corresponding standard errors, and the estimated numbers of persons in the population. These were calculated by applying weights to generalize the answers of the respondents to the population of U.S. civilian, noninstitutionalized adults aged 18 to 49. Statistical tests, such as chi-squares, odds ratios, and corresponding 95% confidence intervals (CIs) were used to examine differences in the distribution of outcomes within demographic and substance use subgroups. Bivariate associations are presented because our primary interest was to identify vulnerable populations rather than build explanatory models using multivariate statistical modeling approaches. However, we also estimated a single multivariable (i.e., adjusted) model using logistic regression. The independent variables included demographics, ADHD status, and drug use history. We present the significance effect of each characteristic after adjusting for potential confounders in the model. All analyses used SUDAAN (Release 9.01) to account for the sampling design by making appropriate use of the calculated sample weights [36]. As this was not a multistage design, no adjustments were necessary to correct the standard errors for the sampling of respondents within strata.

## Results

### Prevalence of lifetime and past-year NMU

An estimated 7.1% of U.S. adults aged 18 to 49 used nonmedically any ADHD medication, which includes both stimulants and nonstimulants, at least once in their lifetime, and 2% did so in the past year (Table 2); estimates were comparable for ADHD stimulants. NMU of short-acting forms of ADHD stimulants was more prevalent than that of long-acting ones, both for lifetime (5.4% for short-acting ADHD stimulants versus 2.1% for long-acting) and past-year (1.6% versus 1.0%) periods. (See the appendix for examples of how survey information was obtained on NMU of short- and long-acting medications). Many of the specific drug estimates for past-year NMU (Table 3) should be interpreted cautiously because of their imprecision, as shown by the magnitude of the standard errors relative to the estimates. Most respondents who reported past-year NMU reported such use on multiple occasions – an estimated 30% of past-year NMUs of ADHD medications used on 1 or 2 days in the past year, and 70% used on 3 or more days.

**Table 3:** Selected past year nonmedical use of prescription and illicit drugs, ADHD diversion study, National Survey of Drug Use and Health, 2005

	**Past Year Use**					
	
	**2005 Diversion**		**2005 NSDUH**			
			
	***n *= 4,297**		***n *= 32,104**			
	**%**^a^	**SE**	**%**^a^	**SE**	***P***^b^	**ES**^c^
**Illicit Drugs**						
Marijuana	14.63	1.23	15.01	0.28	0.76	0.011
Cocaine	2.73	0.44	3.51	0.14	0.09	0.042
Methamphetamine	0.71	0.14	0.80	0.07	0.55	0.010
Supraval^d^	0.09	0.05				
**Prescription stimulants**^e^						
Prescription diet pills^f^	0.81	0.24	n/a			
Ritalin or methylphenidate	0.57	0.14	n/a			
Cylert or pemoline	0.14	0.07	n/a			
Dexedrine, Dextrostat, or Dexampex	0.43	0.24	n/a			
Dextroamphetamine	0.32	0.24	n/a			
**Prescription stimulants**^e^						
Preludin or phenmetrazine	0.06	0.05	n/a			
Any Adderall	0.92	0.13	n/a			
Adderall	0.77	0.13	n/a			
Adderall XR	0.55	0.11	n/a			
Concerta	0.42	0.18	n/a			
Any ADHD stimulants^g^	1.96	0.34	n/a			
**ADHD Nonstimulants**						
Modafanil	0.12	0.05	n/a			
Strattera	0.36	0.24	n/a			
**Any ADHD medications**^h^	**2.01**	**0.34**	**n/a**			
Used 1 or 2 times in past year	0.49	0.17	n/a			
Used 3 or more times in past year	1.50	0.29	n/a			

ADHD = attention deficit hyperactivity disorder; n/a = Estimate not available from the 2005 NSDUH; NSDUH = National Survey of Drug Use and Health.

^a ^Weighted to the U.S. population of noninstitutionalized civilian adults aged 18 to 49.

^b ^*P *value derived from chi-square test in SUDAAN.

^c ^Effect size, determined by the difference in prevalence estimates between the 2005 Diversion Study and the 2005 NSDUH. In the social sciences, an effect size is considered to be "small" when it is about 0.10, "medium" when it is about 0.30, and "large" when it is about 0.50, based on Cohen's d (1988) [32].

^d ^Fictitious drug used to assess reporting bias.

^e ^Nonmedical use.

^f ^Examples given in both studies were amphetamines, Benzedrine, Biphetamine, Fastin, or phentermine. Because some of these medications may be used to treat ADHD (e.g., amphetamines), respondents in the 2005 Diversion Study who reported lifetime nonmedical use of "prescription diet pills" were subsequently asked separate questions about nonmedical use of (a) amphetamines; (b) Benzedrine; (c) Biphetamine; (d) Fastin, or phentermine; or (e)some other prescription diet pill.

^g ^ADHD stimulants referred to any of the following: amphetamines or dextroamphetamine (including any form of Adderall, Biphetamine, Dexedrine, Dextrostat, or Dexampex); any form of methylphenidate or dexmethylphenidate (including Ritalin, Concerta, Methylin, or Focalin); or Cylert or pemoline.

^h ^Nonmedical use of any ADHD stimulants or nonstimulants in the period of interest.

### Past-year NMU in selected population subgroups

Bivariate data in Table 4 indicate that the most vulnerable populations for NMU were those abusing illicit drugs – including methamphetamine (OR = 48.3 for past-year users versus nonusers), cocaine (OR = 26.4), marijuana (OR = 7.3) – and alcohol (binge drinking OR = 5.1). In addition, 69.2% of NMUs reported binge drinking in the past month, and 53.9% used marijuana in the past year, but only 16% were past-year methamphetamine users (data not shown).

**Table 4:** Correlates of past year nonmedical ADHD medication use

	**Sample**	**Unadjusted**^a^		**Adjusted**^a^	
	**%**^b^	**OR**	**95% CI**	**OR**	**95% CI**
**Age**					
18–25	4.34	3.52	1.76–7.08	1.89	0.87–3.64
26–49	1.27	1.00			
**Sex**					
Female	1.66	0.69	0.35–1.36	1.03	0.48–2.22
Male	2.37	1.00		1.00	
**Race/ethnicity**					
White	2.35	1.00			
Nonwhite	1.36	0.57	0.30–1.09	0.59	0.29–1.19
**Education**					
Less than high school graduate	1.21	0.65	0.30–1.42	0.81	0.33–1.95
High school graduate	1.85	1.00		1.00	
Some college	3.26	1.79	0.78–4.09	1.72	0.72–4.13
College graduate	1.41	0.76	0.37–1.55	0.75	0.33–1.71
**Student status (age 18–25)**^c^					
College student	4.57	1.10	0.67–1.81		
Nonstudent	4.18	1.00			
**Insurance**					
Public	2.12	1.00		1.00	
Private	1.69	0.80	0.42–1.51	0.79	0.40–1.58
**ADHD status**					
No dx w/meds lifetime	1.53	1.00		1.00	
Meds in lifetime, but not past year.	8.94	6.31	1.84–21.44	6.04	1.72–21.15
Meds in past year	25.57	22.08	9.53–51.18	9.95	2.55–38.85
**Past-month binge alcohol use**^d^					
No	0.90	1.00		1.00	
Yes	4.46	5.13	2.48–10.63	4.38	1.70–27.42
**Past-year marijuana use**					
No	1.09	1.00		1.00	
Yes	7.41	7.29	3.51–15.16	16.78	6.66–42.27
**Past-year methamphetamine use**					
No	1.70	1.00		1.00	
Yes	45.50	48.27	20.62–112.91	2.00	0.76–5.29
**Past-year cocaine use**					
No	1.33	1.00		1.00	
Yes	26.23	26.37	11.23–61.92	2.73	1.13–6.60

ADHD = Attention deficit hyperactivity disorder; CI = Confidence interval; OR = Odds ratio (1.00 = reference group).

^a ^Unadjusted models are bivariate associations. The adjusted model was a single model that included all the characteristics listed in this table as covariates.

^b ^Weighted to the U.S. population of noninstitutionalized civilian adults, aged 18 to49.

^c ^Student status was not estimated in the adjusted model.

^d ^Defined as having five or more drinks in a single occasion at least once in the past 30 days.

Higher rates of NMU of ADHD medications also were observed for those who self-reported that they had received a legitimate prescription for ADHD medications in the past year (OR = 22.1) or had received medications in their lifetime but not in the past year (OR = 6.3). Although ADHD apparently elevated the risk of NMU, 1.9 million of the 2.6 million persons aged 18 to 49 (73%) who participated in NMU had never been diagnosed and treated pharmacologically for ADHD. Further, fewer than 5% of past-year users who used ADHD medications nonmedically were prescribed ADHD medications in that period.

NMU of ADHD medications was more prevalent among young adults aged 18 to 25 (4.3%) than among adults aged 26 to 49 (1.3%). No significant differences were observed in key demographic subgroups, including gender, race, student status, and insurance status.

Similar to the bivariate models, in the multivariate logistic regression model, the likelihood of past-year NMU of ADHD drugs was higher for past-year marijuana users and binge drinkers than for their nonusing counterparts. Receipt of ADHD medications in the past year or lifetime also remained significantly associated with NMU of ADHD drugs in the multivariate model. However, the association for past-year methamphetamine use became nonsignificant in the multivariate model, suggesting that the strong bivariate association between methamphetamine use and NMU of ADHD drugs may be accounted for largely by other demographic, psychosocial, and substance use characteristics. This could be due either to confounding in which NMU and methamphetamine use are related to a third demographic or substance use factor, or a mediational pathway in which the exogenous factors influence methamphetamine use, which influences NMU of ADHD medications. The effect of age also became nonsignificant in the multivariate model, suggesting a possible mediational pathway in which age may influence ADHD symptoms and substance use behaviors such as binge alcohol use and marijuana or cocaine use. In turn, other drug use and ADHD symptoms may influence the likelihood of past-year NMU of ADHD medications.

### Sources of diverted medications and motivations for NMU

**Figure 1:**
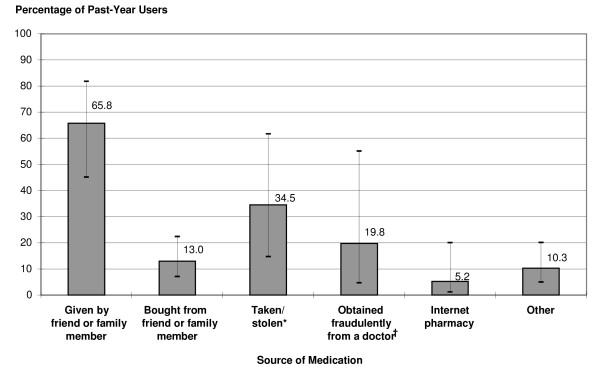
**Sources of diverted adhd medications among past-year nonmedical users**. Percentages of past-year nonmedical users of ADHD medications aged 18 to 49 who were never told that they had ADHD or were never prescribed medications specifically for ADHD and who were estimated to have obtained diverted medications in specific ways (including error bars). Estimates are not mutually exclusive. Bars represent 95% confidence intervals. ADHD = attention deficit/hyperactivity disorder. * Stolen from friends, family members, or other sources. ^† ^Obtained fraudulently by mispresenting symptoms or presenting to a physician who "didn't ask too many questions."

Figure 1 presents findings on sources of diverted ADHD medications for the NMUs who reported never having an ADHD diagnosis and prescription. The 95% confidence intervals also are presented to show the precision of the estimates. The most common source among this group was a friend or family member (66%) who gave away some of their prescription. More than 34% of NMUs had taken or stolen medications from friends, family, or other sources. However, physicians also were a significant source of diverted medications for this group, with nearly 20% of NMUs having obtained fraudulent prescriptions by fabricating symptoms or presenting to doctors who were known to "not ask too many questions." An estimated 5% of NMUs without a legitimate need for a prescription procured their medications over the Internet.

**Figure 2:**
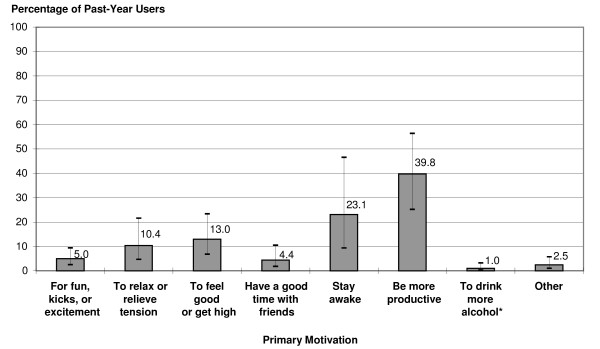
**Primary motivation for nonmedical adhd use among past-year users**. Percentages of past-year nonmedical users of ADHD medications who were estimated to have specific primary motivations for misusing ADHD medications (including error bars). Percentages sum to less than 100 because of missing data from respondents who were not sure of their answer or refused to answer. Bars represent 95% confidence intervals. ADHD = attention deficit/hyperactivity disorder.* Response option available only to those who reported past 12-month alcohol use.

Principal motivations among past-year NMUs were productivity (40%) and staying awake (23%) (Figure 2). Use principally to "get high" (13%), for tension relief (10%), or for fun, kicks, or excitement (5%) also were noted. About 1% of past-year NMUs used ADHD medications principally to facilitate alcohol use.

### Past-year NMU of ADHD medications in combination with selected drugs

**Figure 3:**
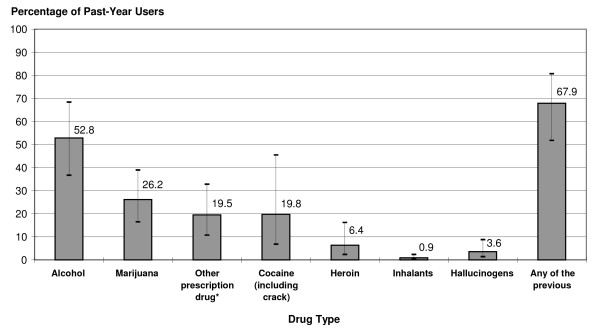
**Use of alcohol or selected other drugs in combination with nonmedical use of adhd medications in the past year**. Percentages of nonmedical users aged 18 to 49 in the U.S. civilian noninstitutionalized population who used any of the listed drugs at the same time or within a couple of hours of using ADHD medications nonmedically in the past year (including error bars). Estimates are not mutually exclusive. Bars represent 95% confidence intervals. ADHD = attention deficit/hyperactivity disorder.* Examples given included painkillers, tranquilizers, or sleeping pills. Use in combination (either at the same time or within a few hours) with ADHD medications could include legitimate prescription use or nonmedical use of other medications.

Among those who had used ADHD medications nonmedically in the past year, 68% also used alcohol, other prescription medications, or illicit drugs at the same time or within a couple of hours of their nonmedical ADHD medication use (Figure 3). More than half (53%) of past-year NMUs drank alcohol while using these medications, and 26% used marijuana. Nearly one in five past-year NMUs aged 18 to 49, had used ADHD medications in combination with cocaine at least once in the past year.

## Discussion

Despite media attention on the nonmedical use of prescription ADHD medication [37], our understanding about the characteristics of NMU remains relatively underdeveloped compared to other substances such as alcohol and tobacco. This study contributes new data on a variety of issues related to NMU that provide insight into the patterns of NMU of prescription ADHD medications and associated risk factors in the United States. However, results from our study are not directly comparable to other data sources given that our study focuses on specific types of ADHD drugs rather than on stimulants in general. Most directly comparable is the study conducted by Kroutil and colleagues [7] using the 2002 NSDUH. Based on those data, the past-year prevalence of NMU of ADHD stimulants was higher among young adults aged 18 to 25 (1.3%). This estimate is lower than our estimate of 4.3% for those aged 18 to 25 for at least two reasons. First, the current study covered a larger range of stimulant medications, including medications such as Adderall, than the NSDUH. Also, the Kroutil et al. [7] study estimated NMU of ADHD medications for persons who had not misused other stimulants; that likely underestimated the prevalence of any NMU of ADHD medications.

Although not directly comparable because the estimates are based on only stimulants, the 2005 NSDUH estimated that 3.6% of persons aged 18 to 25 had used any stimulant nonmedically in the past year. We also estimated the past-year prevalence from the public use files of the National Epidemiologic Survey on Alcohol and Related Conditions [38], which was found to be 1.7% among those aged 18 to 25. Follow-up data for young adults from the 2005 Monitoring the Future survey indicated that 5% to 7% of young adults aged 19 to 24 used amphetamines "not under a doctor's orders," and 2.9% to 5.5% did so for Ritalin [39]. Results from the College Alcohol Study revealed that the rate of past-year NMU of prescription stimulants among college students was 4.1% [40], which was similar to the 4.6% reported by students aged 18 to 25 in the current study.

Inspection of the prevalence rates from various community-based investigations revealed that, even among representative probability surveys, there is a wide degree of variation, which may be due to differences in methodology. For example, the NSDUH uses a definition of NMU based on use without a doctor's prescription or "only for the experience or feeling it caused." This final definition resulted from methodological testing [23,41] to simplify the concept of "nonmedical" use for NSDUH respondents, some of whom are as young as 12. This definition has been criticized for leaving open the possibility that legitimate use may be reported by mistake [42]. However, differences in prevalence estimates between surveys such as the NSDUH and National Epidemiologic Survey on Alcohol and Related Conditions (NESARC) also may be related to differences in mode of administration; NSDUH questions about drug use are self-administered, but NESARC is administered directly by interviewers. Other research suggests that respondents may underreport sensitive behaviors if asked directly by interviewers [43]. Nevertheless, findings on the correlates of NMU of ADHD medications from this study were largely consistent with previous research, providing a degree of confidence in our methods. In particular, we observed higher rates of NMU among young adults aged 18 to 25 than among older age groups, which parallels other national data [44,45].

There also is significant interest in the rates of NMU among students. Recent studies suggest that young adults, and particularly students, may account for much of the NMU of prescription stimulants. For example, Herman-Stahl et al. [9] found in analyses of NSDUH data that college students were at a greater risk of NMU of prescription stimulants than their noncollege peers. Further, previous college enrollment or graduation also was a significant risk factor, suggesting that NMU of stimulants in general may persist beyond college. However, the current study found no statistically significant (*P *< .05) differences in the rates of NMU of ADHD medications between young adult students (4.6%) and young adult nonstudents (4.2%), although we considered a broader range of medications and focused on ADHD drugs rather than on prescription stimulants in general. It also should be noted that our definition of student status included technical students and did not distinguish between full-time and part-time students. Thus, future studies should probe for differences in NMU by student enrollment status, including full-time, part-time, public, private, technical, and community college.

Another important contribution of this study involves examination of the patterns of polydrug use among NMUs of ADHD medications. Binge alcohol use (69%) and marijuana use (54%) were fairly common among past-year NMUs, which also has been found in a previous study [9]. Although most ADHD medications are classified as stimulants, only 16% of past-year NMUs of ADHD medications also were past-year methamphetamine users. In contrast, 46% of methamphetamine users commonly misused prescription ADHD medications. One possible explanation for this latter finding is that methamphetamine users may engage in NMU of ADHD stimulants as a substitute when methamphetamine is unavailable. However, additional studies, using items that capture aspects of substitution among methamphetamine users, are needed to specifically test this assertion. Overall, NMU of ADHD medications by methamphetamine users appears to constitute a relatively small portion of the overall diversion of ADHD medications in the United States, although use of methamphetamine is a powerful risk factor for NMU.

A significant strength of this study was that we also asked about the concomitant use of ADHD medications with other drugs and alcohol. We observed that approximately 68% of past-year NMUs had used at least one other substance either at the same time or within a couple of hours of NMU of ADHD medications. Findings from McCabe et al. [11] using a sample drawn from a single university showed that most polydrug use involving alcohol and prescription drugs occurred simultaneously rather than on separate occasions. The high rate of alcohol use in combination with NMU of ADHD drugs is alarming in these studies, given that ADHD stimulants can counteract the depressant effects of alcohol. This leaves open the possibility that persons who drink alcohol may be able to prolong their drinking by also using stimulants, thereby increasing their risk of alcohol poisoning. Findings from the Drug Abuse Warning Network [46] indicate that over 2% of all emergency department visits involving alcohol also involved prescription stimulants [46]. We also found that nearly one in five NMUs of ADHD medications had used them in combination with cocaine, which may increase the risk of adverse cardiac events, given known risks associated with cocaine use [47] and recent concerns about potential cardiovascular risks associated with specific ADHD medications [48]. The issue of co-occurrence is particularly germane because alcohol and other drugs have the potential to affect the synaptic transmission produced by stimulant and nonstimulant ADHD medications, thus affecting regulation and causing adverse immediate and long-term health consequences [49-51].

Recent research has found elevated rates of substance use among those with ADHD [16,52]. Similarly, findings from our study indicated that those who self-identified that they had been diagnosed by a physician with ADHD and had been prescribed medication were at increased risk for NMU of ADHD medications. However, NMU by this subpopulation constitutes a relatively small proportion (less than 5%) of the total past-year NMU in the United States. Further, interpretation of the association between nonmedical use of ADHD medications and a history of pharmacologic treatment for ADHD requires caution. Our data are cross-sectional and preclude causal inferences, such as concluding that prescribing medications with potential abuse liability increases the likelihood of NMU. Accumulating evidence from prospective research also suggests that treatment of ADHD with stimulants in and of itself does not confer additional liability to NMU of ADHD stimulants or other substances [53,54].

One possible explanation for the relation between NMU and an ADHD diagnosis or receipt of medications is that some patients receiving pharmacological treatments may be undermedicated or noncompliant with their prescribed therapeutic dosage. Consequently, they may take excessive quantities to self-medicate or overcompensate for earlier noncompliance because of an increase in the quantity and severity of untreated symptoms. However, undermedication seems a less plausible explanation for NMU in this study, as the question wording for NMU primes the respondent to answer about use beyond the therapeutic value of the drug as prescribed by a physician or medical professional (see the appendix). However, productivity (39.8%) was a commonly endorsed motivation for NMU for all respondents, regardless of ADHD status. Thus, it is possible that a proportion of NMUs who are taking ADHD medications to be more productive may be self-medicating ADHD symptoms. It is also possible that a large proportion of those whose primary motivation for NMU is productivity may actually meet the criteria for ADHD, but have not received a formal medical diagnosis. In contrast to self-medication, another prominent subgroup of NMUs – nearly 30% – could be considered recreational users whose principal motivations were tension relief, euphoria, or thrill-seeking. Another possible explanation linking ADHD and NMU is that an additional set of vulnerability factors linked to NMU, such as co-occurring psychiatric disorders, may increase the likelihood of NMU of ADHD medications. We are conducting additional analyses to examine the complex issues related to treatment status, ADHD symptoms, NMU, and motivations for use. Thus, additional research is needed with persons being treated pharmacologically for ADHD to compare NMU between those with and without psychiatric comorbidity to identify the combination of risk factors that can account for vulnerability to NMU.

Overall, these findings should be interpreted in light of several considerations. First, the validity of self-reported drug use among Internet respondents depends on their willingness to answer truthfully about drug use in that data collection mode and their ability to recall use of specific drugs within designated time frames. To aid in recall, our online survey questionnaire supplied respondents with reference dates to establish recall periods such as the past 12 months and, where possible, made pictures of medications available to respondents. We have also shown that our findings are consistent with data from a large national probability survey (NSDUH), as well as with findings from other studies on NMU. Confidence in our findings is also supported by the low prevalence of self-reported NMU of the fictitious drug Supraval (lifetime: 0.11%; past year: 0.09%), suggesting that respondents were able to discriminate between real and fictitious drugs. Substance use data have been shown to be statistically comparable between data collection modes involving mail surveys and the Internet [55], as well as between the Internet and telephone [56]. HI also conducts routine screening of Internet IP addresses and conducts follow-ups with members to ensure that each participant is not using multiple e-mail accounts to opt into the panel. For security reasons, each panelist is also assigned a unique and confidential ID to ensure privacy. Together, these findings strengthen our confidence in the accuracy of the self-reports and use of the Internet recruitment design. Although the convergence of our findings with those from other studies does not rule out misreporting of drug use in our data, it does not suggest a markedly greater misreporting problem in our study than in others. Nevertheless, the possibility of a mode effect does remain [57], and researchers should be sensitive to its presence by comparing estimates from other data sources to check for reliability of the data.

Second, concerns about selection bias may arise from the low overall number of responses relative to the number of e-mail invitations that were sent and from our use of a nonprobability Internet survey to estimate NMU. With regard to the numbers responding, it should be noted that this method differs from other survey research methods, in that the current study did not employ numerous follow-up attempts or refusal conversion efforts. Further, bias is defined as the difference between the sample estimate and the true population estimate. If the results from other nationally representative data systems (e.g., NSDUH, NESARC) are used as the "true" gold standard population estimates, then comparing our results as the sample estimates to those found in other studies as the population estimates reveals no appreciable degree of bias. In addition to the studies noted above (e.g., NESARC, MTF), a direct comparison of estimates between the current study and the 2005 NSDUH, the most methodologically parallel study, for variables that were not used in our weighting procedures suggests that we reduced to a large degree the potential biases resulting from the sample selection for the online survey. In additional analyses not shown, we also estimated parallel regression models using the 2005 NSDUH and our Internet sample. For the outcomes of past-year marijuana use and past-month binge alcohol use, we observed high consistency in the estimated odds ratios for common demographic characteristics measured in both studies. The primary difference in the estimates for these variables was that the width of the confidence interval around each estimate was wider in the Internet study than in the NSDUH. This could be expected given the differences in the sample sizes between the NSDUH (N = 32,104) and our study (N = 4,297). If bias existed in the propensity score algorithm used to generate the sample weights, we would have expected to observe differences in the estimates in addition to the confidence intervals. Epidemiologic studies of NMU of ADHD drugs can suffer from other biases inherent to sample selection, such as samples drawn from clinical populations, local or regional samples, or samples of demographic subgroups that are accessible for surveys (e.g., students). Therefore, we believe that our study adds to the base of knowledge about NMU of ADHD medications through its national focus and depth of coverage of relevant issues.

We note that the value of the Internet panel design is meant to complement, not replace a national probability study. Internet surveys may be a promising approach for conducting formative research or gathering information on a focused topic for less time and expense than other survey methods. Internet panels are burgeoning in many countries around the world, and this study design is appearing in the research literature with greater frequency. For example, Schlenger et al. (2002) [58] used such an approach to examine the psychological reaction to the terrorist attacks of September 11, 2001, within weeks after the event. Another study by West et al. (2006) [59] used the Harris Interactive Online Poll, a panel with several million members in 125 countries, to examine patterns of smoking cessation. Their combined sample consisted of 2,009 participants from the United States, Canada, the United Kingdom, and France. They compared characteristics of cigarette smokers from a standing Internet panel who were selected for the study to characteristics of smokers in national data systems in each respective country and found a high degree of correspondence on major demographic characteristics. In particular, the rapidity with which Internet surveys can be conducted may open new opportunities for cross-national studies of NMU of prescription medications. Under careful methodological rigor, this approach may be favorable to a small face-to-face survey or a large telephone survey. Nevertheless, results from an Internet design should be regarded as preliminary estimates that should be replicated using national probability samples.

## Conclusion

Given these considerations, this research has important implications for addiction research and policy. Future studies, perhaps using longitudinal designs, should consider examining factors that may be important for the initiation of NMU, progression to abuse or dependence, and transitions to other drugs, such as methamphetamine. These studies might examine the relevant contributions of access to medications, attitudes toward use, and motivations for use as they relate to these varying trajectories over time. Additional studies may also consider examining the use of specific ADHD medication formulations, such as long-acting versus short-acting medications, as well as the prevalence of NMU formulations in relation to different prescribing practices across various geographic areas.

In terms of public policy, regulatory options to discourage diversion may be limited. In the United States, for example, many of these medications are already under the highest level of Federal regulation for legally approved controlled substances (i.e., Schedule II under the Controlled Substances Act). Nevertheless, about 20% of NMUs obtained diverted ADHD medications directly from physicians, with a much smaller percentage (5%) obtaining them from Internet pharmacies. Thus, further education may be needed to aid physicians in recognizing when patients are attempting to obtain ADHD medications fraudulently. Legislation also may be needed to create additional prescription drug monitoring programs at the state level or stricter penalties for lax prescribing practices for ADHD medications.

Formulation of medications also plays a significant role in drug abuse [60]. Therefore, decisions about which medications are included in insurance providers' formularies could affect ADHD medication prescribing practices and could decrease the availability of those ADHD medications with greater abuse liability.

Consistent with other research, however, this current study showed that persons with prescriptions for ADHD medications were the leading source of diverted medications. For example, a study of ADHD stimulant diversion among college students at a Midwestern university found peers to be the most common sources of diverted prescription ADHD stimulants [61]. Findings from the 2005 NSDUH also reported that the most recent stimulants used nonmedically were provided by friends or relatives [14]. About 35% of NMUs in our study who did not have a prescription of their own were estimated to have stolen ADHD medications. Thus, some people who have a prescription to treat ADHD may unknowingly be supplying ADHD medications that are taken without their permission.

Because friends and family are important sources of diverted medications, education of patients with prescriptions for ADHD medications will be important in reducing the risk of NMU and deterring informal distribution to family and friends. As such, intervention programs should be developed to educate patients regarding the potential for diversion, whether the medications are intentionally shared or taken without the patient's knowledge.

Finally, the use of this Internet survey methodology may be relevant to policy makers with limited budgets who are seeking timely information on a relatively low-prevalence phenomenon in a target population. Again, this methodology would not supplant national probability surveys. If an external data source exists for use as a benchmark, however, this data collection method could provide policy makers and planners with usable information in a relatively short amount of time.

## Competing interests

There are no financial competing interests. The design, methods, and analysis for this study were conducted by RTI International, with funding from Eli Lilly and Company, which is a pharmaceutical manufacturer. RTI received the data collection and analysis contract through a competitive bid. Except for the fourth author, all coauthors were employees of RTI International (RTI), an independent nonprofit research firm. Researchers at RTI had full access to all of the data in the study and exercised full control over the interpretation and reporting of the results. Study design and data collection for the NSDUH were done by the Substance Abuse and Mental Health Services Administration within the U.S. Department of Health and Human Services. However, the views expressed in this paper do not necessarily reflect official policies of the U.S. Department of Health and Human Services.

## Authors' contributions

SPN led the team in the analysis and drafted the paper. LAK conceptualized the analytic plan, study design, and wrote sections of the paper. RLW conducted the analyses. DLB assisted in study design. All authors reviewed drafts of the manuscript and provided substantive and methodological input.

## Appendix

The wordings of questions about lifetime NMU are shown below. Respondents used "radio" buttons to indicate their use or nonuse of each drug. Respondents were required to enter an answer for every drug (which could include "Not sure" or "Decline to answer") before the interview would allow them to move on. The following is an excerpt from the survey:

"Now we have some questions about drugs that people are supposed to take only if they have a prescription from a doctor.

We are only interested in your use of a drug if:

• the drug was not prescribed for you, or

• you pretended to have symptoms (or worse symptoms) to get a prescription, or

• you took the drug only for the experience or feeling it caused.

People sometimes take these medications to lose weight, to stay awake, for attention deficit disorders, or other reasons. We are not interested in the use of "over-the-counter" drugs such as Dexatrim, No Doz, Benadryl, or Nytol that can be bought in drug stores or grocery stores without a doctor's prescription.

The next pages contain pictures of some of the drugs we will be asking you about. Please look carefully at the pictures of these drugs before you answer the next questions.

[DRUG PICTURE SCREENS FOLLOWED] Please select the "NEXT" button at the bottom of your screen to continue with the survey.

To identify past-year NMU, respondents were asked specifically, "How long has it been since you *last *used the drugs shown below when they were not prescribed for you or you took them only for the experience or feeling they caused?" Respondents were shown only drugs that they reported ever using nonmedically. Valid response choices were (1)within the past 30 days – that is, since MONFILL (where "MONFILL" was calculated from the interview date to define the 30-day reference period); (2)more than 30 days ago but within the past 12 months; (3)more than 12 month ago but within the past 3 years; and (4)more than 3 years ago. Respondents who reported that they last used an ADHD medication nonmedically "in the past 30 days" or "more than 30 days ago but within the past 12 months" were defined as past-year NMUs.

During the past 12 months, what is the main reason you used [this drug/these drugs] without a doctor's prescription or only for the experience or feeling [it/they] caused?

1) For fun, kicks, or excitement

2) To relax or relieve tension

3) To feel good or get high

4) To have a good time with friends

5) To help yourself be more productive

6) To stay awake

7) To fit in with a group you like

8) (IF PAST YEAR ALCOHOL USE REPORTED) To be able to drink more alcohol

9) Some other reason

(NOTE: Respondents not asked to specify the "other" reason.)

98 Not sure

99 Decline to answer

Questions on self-reported ADHD diagnosis and medications are shown below. Questions AD01 and AD02 appeared fairly early in the survey, but after questions DR01 through DR13 about lifetime nonmedical use of specific prescription medications. Question ADRXYR appeared toward the end of the survey, after all questions about nonmedical use of ADHD drugs, nonmedical use of other stimulants, and problems associated with nonmedical use of prescription drugs.

These next questions are about attention-deficit/hyperactivity disorder, also known as ADD or ADHD.

AD01. Did a doctor or other medical professional ever tell you that you had ADD or ADHD?

1 Yes

2 No

8 Not sure

9 Decline to answer

AD02. (If AD01 = 1) Did a doctor or other medical professional ever prescribe any medications for your ADD or ADHD?

1 Yes

2 No

8 Not sure

9 Decline to answer
